# Research on functional constipation with anxiety or depression: a bibliometric analysis

**DOI:** 10.3389/fpsyt.2025.1607297

**Published:** 2025-06-18

**Authors:** Xiaoqin Li, Qiang Lei, Jiao Xie, Fang Li, Jing Liu, Yuelai Chen, Qiangjian Mao

**Affiliations:** 1 Clinical Medicine College, Jiangxi University of Chinese Medicine, Nanchang, Jiangxi, China; 2 Acupuncture and Moxibustion Massage College, Jiangxi University of Chinese Medicine, Nanchang, Jiangxi, China; 3 Sleep Medical Center, Affiliated Hospital of Jiangxi University of Chinese Medicine, Nanchang, Jiangxi, China; 4 Sleep Medical Center, Jiangxi Hospital of Longhua Hospital Shanghai University of Traditional Chinese Medicine, Nanchang, Jiangxi, China; 5 Acupuncture and moxibustion Department, Affiliated Hospital of Jiangxi University of Chinese Medicine, Nanchang, Jiangxi, China

**Keywords:** functional constipation, anxiety, depression, bibliometric, CiteSpace

## Abstract

**Background:**

Although the phenomenon of functional constipation (FC) that accompanies anxiety or depression has been extensively investigated worldwide, no bibliometric studies are available in this regard. This study therefore aimed to analyze the current status and extent of research and areas of interest in the study of FC with anxiety or depression.

**Methods:**

Data from studies on FC with anxiety or depression, that were performed between 2003 and 2024, were retrieved from the Web of Science Core Collection database. Data regarding the annual number of publications, authors, countries, and references were assessed using CiteSpace v6.3.R1 (64-bit) and Microsoft Excel, and those pertaining to keywords and cited authors were evaluated using VOSviewer 1.6.20. The co-occurrence and clustering functions were then used to generate visual knowledge maps.

**Results:**

The overall annual publication volume demonstrated an upward trend between 2003 and 2024; this was indicative of promising research prospects. The 427 publications identified included 6 types of papers, among which original research articles represented the highest proportion (357 [83.61%] articles published across 200 journals). *Neurogastroenterology and Motility* had the highest publication volume (30 articles, 7.02%). The United States of America had published most of the papers (135 articles, 31.61%) on the topic. Harvard University was the research institution with the most published papers (21 articles, 4.92%), and Michel Bouchoucha had authored the highest number of articles (13 articles, 3.04%).

**Conclusion:**

Future studies in the field of basic medicine need to determine the etiology and pathogenesis of FC with anxiety or depression; in particular, they need to evaluate the role of opioid drugs as a key etiological factor. The role played by the brain-gut axis also warrants investigation. From the clinical perspective, studies need to focus on evidence-based medicine; particular emphasis needs to be placed on randomized double-blind controlled trials with stringent quality control, high-quality meta-analyses, and evaluation of questionnaires and scales. Treatment techniques need to be explored in greater detail; in this context, it is recommended that fecal microbiota transplantation and biofeedback therapy are adopted in the clinic. Furthermore, Patients with FC, especially those with a history of anxiety or depression, tend to have overlapping dyspepsia symptoms.

## 1 Introduction

Functional constipation (FC) is a common functional bowel disorder, which is caused by non-organic causes and is characterized by continuously difficult, incomplete, or infrequent defecation (1, 2). It is a functional intestinal disease that does not conform to the diagnostic criteria of irritable bowel syndrome (3). Rapid social transition and changes in the diet structure and lifestyle have led to an increase its incidence (4). However, prevalence rates differ across different regions of the world; this may be related to the environment, diet, living habits, culture, diagnostic criteria, and other factors (5–7). Studies show the average global prevalence rate to be 15% (8). In the regional context, a systematic review found the prevalence rate in North America to range from 1.9% to 27% (9). A meta-analysis reported the average prevalence rate in South America, Northern and Southern Europe, the Middle East, and Southeast Asia to be 18%, 16%, 14%, and 11%, respectively (10). FC is a multi-factorial disease with complex pathophysiological mechanisms, which can be related to several factors including the diet, drugs, mental illnesses, or neurological diseases, among others (11).

Studies have shown FC to be associated with mental disorders such as anxiety and depression (12); FC can give rise to anxiety or depression, and vice versa (13, 14). In addition, the severity of constipation has been found to be directly proportional to the clinical severity of anxiety and depression (15). Notably, anxiety is mainly manifested by continuous worry and fear, whereas depression is a mental disease mainly characterized by low mood. Patients with depression usually demonstrate characteristics of anxiety disorders, and vice versa (16). Although the pathogenesis of FC with depression or anxiety remains unclear, currently accepted theories involve the microbiome-mediated bidirectional communication model which is based on the concept of the “brain-gut-microbiome axis (14, 17, 18).”

Bibliometrics refers to the use of statistical and mathematical methods to analyze published literature (such as books, journal articles, and datasets) and their related metadata (such as authors, keywords, citations, and abstracts); this helps to identify hidden associations and describe or display any inter-relationships (19, 20). In addition to objectively presenting the current research status, bibliometrics reveals the developmental trends and extent of research in the specific field (21, 22). It has therefore emerged as an important evaluation tool across various fields worldwide.

To the best of our knowledge, bibliometric analyses on the relationship between FC and anxiety or depression are lacking. This study therefore analyzed the available literature on FC with anxiety or depression, that was published between 2003 and 2024; the aim was to comprehensively evaluate the current status of global research, developmental trends, and hotspots and frontiers in this field.

## 2 Materials and methods

### 2.1 Sources of literature

**Figure 1:**
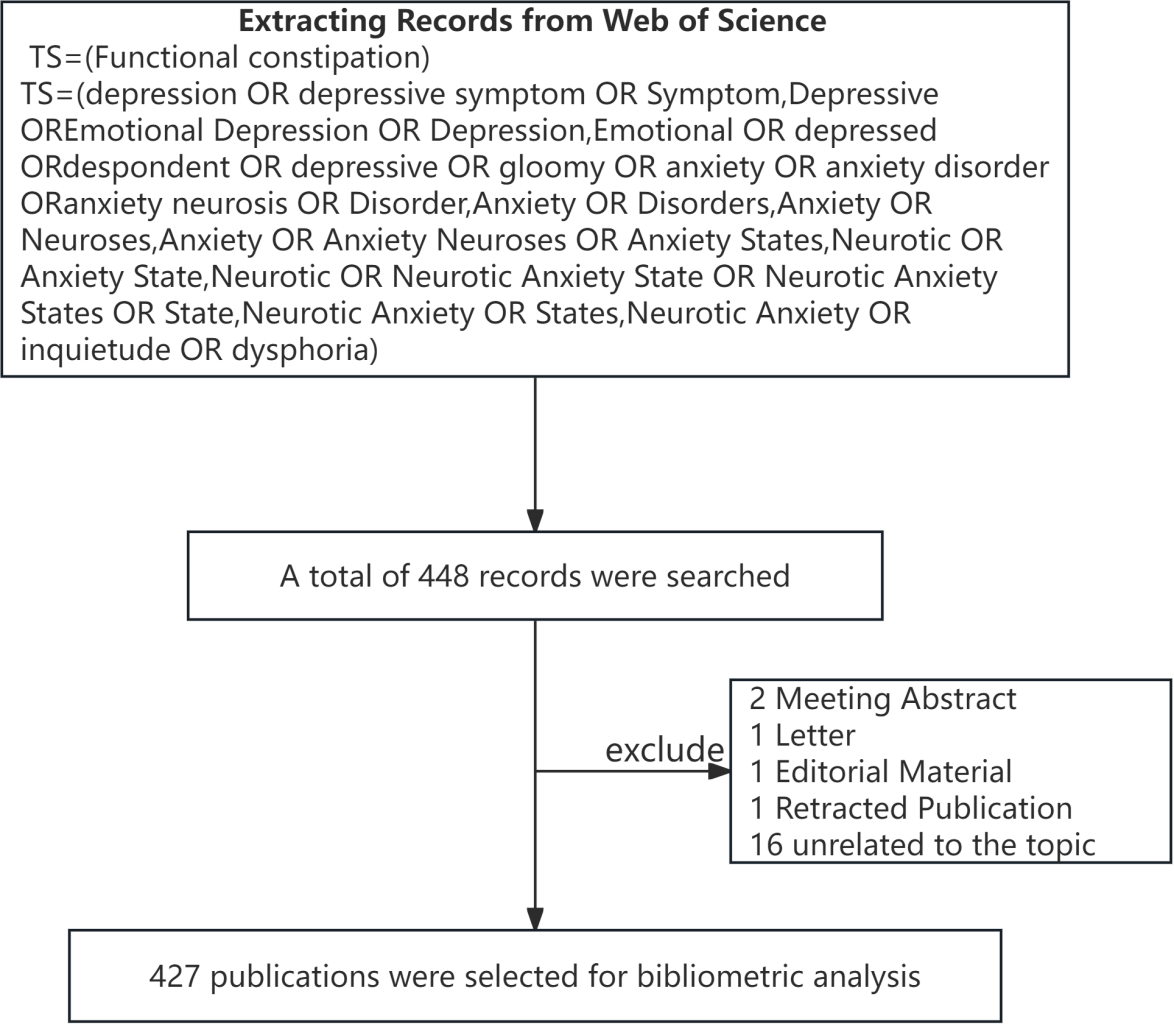
Flow chart of the study.

To avoid omitting any relevant literature, synonyms were first identified from the Medical Subject Headings thesaurus of the PubMed database. The synonyms of anxiety and depression were found to include the following terms: “depression,” “depressive symptom,” “Symptom, Depressive,” “Emotional Depression,” “Depression, Emotional,” “depressed,” “despondent,” “depressive,” “gloomy,” “anxiety,” “anxiety disorder,” “anxiety neurosis,” “Disorder, Anxiety,” “Disorders, Anxiety,” “Neuroses, Anxiety,” “Anxiety Neuroses,” “Anxiety States, Neurotic,” “Anxiety State, Neurotic,” “Neurotic Anxiety State,” “Neurotic Anxiety States,” “State, Neurotic Anxiety,” “States, Neurotic Anxiety,” “inquietude,” and “dysphoria.” No synonyms were found for FC. The Web of Science Core Collection database was searched using the identified synonyms. To ensure authenticity and reliability of the study, there were no restrictions on the language and type of published literature; in addition, the retrieval time was limited between January 1, 2003 and December 31, 2024. We retrieved a total of 448 articles (**Table 1**) and imported the data into CiteSpace; no duplicate articles were found. Some incomplete or irrelevant articles were removed; these included 2 meeting abstracts, 1 letter, 1 editorial article, 1 retracted publication, and 16 articles unrelated to the topic. Finally, 427 articles were included for quantitative analysis (**Figure 1**). The retrieval was performed independently by two authors, namely, Qiang Lei and Jiao Xie, and any disputes were resolved by Qiangjian Mao. The Web of Science database was accessed from the library of the Jiangxi University of Chinese Medicine.

**Table 1:** Search queries.

Set	Results	Search Query
#1	5025	TS=(Functional constipation)
#2	638023	TS=(depression OR depressive symptom OR Symptom, Depressive OR Emotional Depression OR Depression, Emotional OR depressed OR despondent OR depressive OR gloomy OR anxiety OR anxiety disorder OR anxiety neurosis OR Disorder, Anxiety OR Disorders, Anxiety OR Neuroses, Anxiety OR Anxiety Neuroses OR Anxiety States, Neurotic OR Anxiety State, Neurotic OR Neurotic Anxiety State OR Neurotic Anxiety States OR State, Neurotic Anxiety OR States, Neurotic Anxiety OR inquietude OR dysphoria)
#3	448	#1 AND #3

### 2.2 Analysis tools

CiteSpace and VOSviewer are two powerful software tools with complementary advantages (23). The former was developed by Professor Chen Chaomei of Drexel University in the United States (24); it can be used to perform multivariate, time-sharing, and dynamic visualization analysis of data and draw corresponding visualization charts (25). As it has been mainly designed to analyze emerging trends in the research field, it is widely used for bibliometric analyses and data visualization (26). VOSviewer is a free Java-based software application that was developed in 2009 by Van Eck and Waltman of Leiden University in the Netherlands (27). It offers certain advantages including the ability to process large-scale data, a more accurate clustering algorithm, and visually appealing graphs that are easy to interpret (28, 29). Concomitant use of CiteSpace with VOSviewer software can allow superior evaluation of the developmental trends in a certain field. It can also help in the visual analysis of relevant information by establishing a corresponding knowledge map (30), which may help identify more valuable research hotspots and directions and provide a reference for further development in this field (31).

In this study, CiteSpace v6.3.R1 (64-bit) and Microsoft Excel software were used to draw the distribution maps for the annual publication volume, authors, countries, institutions, and references. VOSviewer 1.6.19 software was used to draw the distribution maps of cited authors and keywords.

## 3 Results and discussion

### 3.1 Analysis of the annual volume of publications

**Figure 2:**
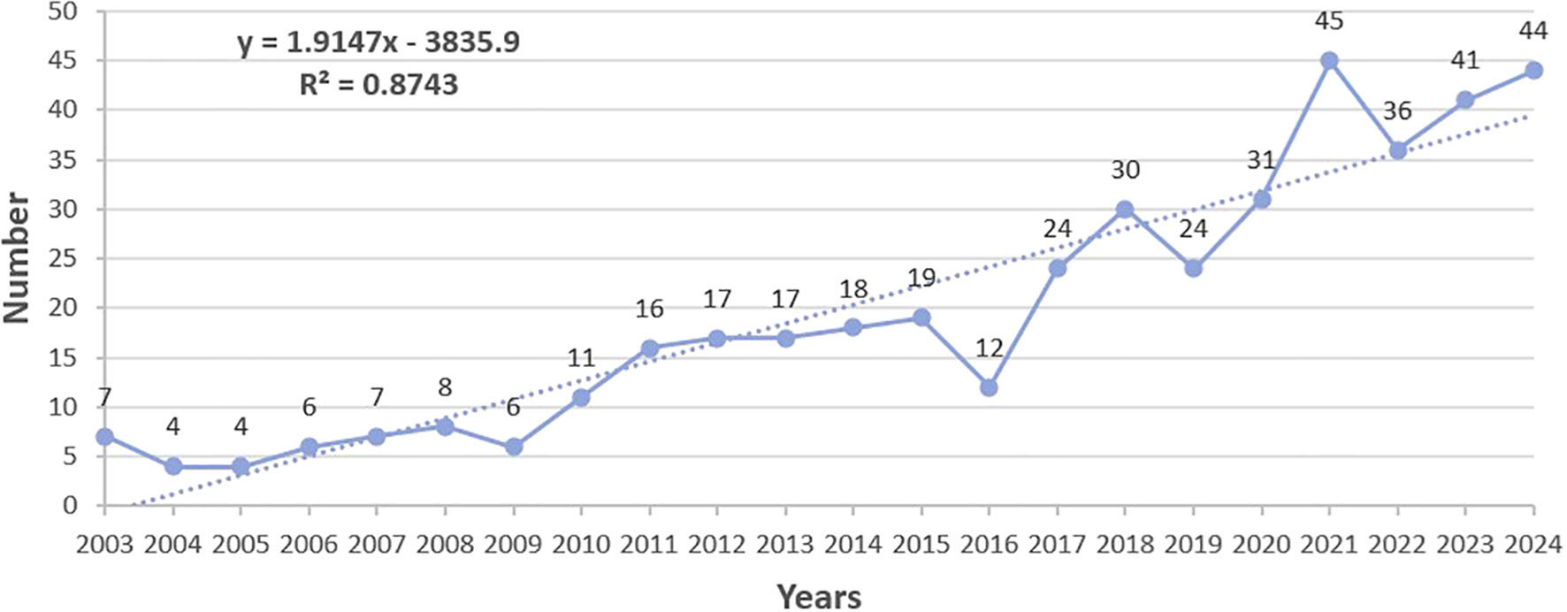
Annual number of publications on FC with anxiety or depression between 2003 and 2024.

Excel software was used to draw the distribution map of number of publications each year; this tool was used as it can reflect temporal trends in development and changes in a field to a certain extent (**Figure 2**). The number of articles related to FC with anxiety or depression fluctuated slightly between 2003 and 2024; although an overall upward trend was observed, the increase has become more pronounced especially in the last five years. High reliability of the trend line was verified by calculating the slope (y = 1.9147x - 3835.9; R^2^ = 0.8743). The quantity of published articles peaked in the year 2021 (45 articles, 10.53%). The findings suggest that the topic of FC with anxiety or depression has attracted increasing attention from researchers, and shows a favorable developmental trend.

### 3.2 Analysis of journals and cited journals

A total of 427 publications were classified into 6 groups based on their type. Articles (357, 83.61%) represented the predominant type of publication, followed by reviews (54, 12.65%), proceeding papers and early access articles (7, 1.64%), and data papers and early access reviews (1, 0.23%), in that order (**Table 2**). These papers were published across 200 journals; the highest number of articles were published in the Journal of *Neurogastroenterology and Motility* (30, 7.02%). This was followed by the *Journal of Neurogastroenterology and Motility* (12, 2.81%), *Clinical Gastroenterology and Hepatology* and *World Journal of Gastroenterology* (9, 2.11% each), and *Journal of Gastroenterology and Hepatology* (8, 1.87%), in that order; the top 10 journals are shown in **Table 3**. Based on data from Journal Citation Reports 2023 (of the American Institute of Scientific Information), *The Lancet* was found to have the highest impact factor (98.4 in 2023) among these journals.

**Table 2:** Literature types related to FC with anxiety or depression.

Rank	Type	Counts (%)	Rank	Type	Counts (%)
1	Article	357 (83.61%)	4	Early access	7 (1.64%)
2	Review	54 (12.65%)	5	Data Paper	1 (0.23%)
3	Proceeding paper	7 (1.64%)	6	Early access review	1 (0.23%)

**Table 3:** Top 10 journals and publications related to FC with anxiety or depression.

Rank	Publications	Journal	IF(2024)	Rank	Publications	Journal	IF (2024)
1	30	Neurogastroenterology and Motility	3.5	6	8	Medicine	1.3
2	12	Journal of Neurogastroenterology and Motility	3.3	7	8	Plos One	2.9
3	9	Clinical Gastroenterology and Hepatology	11.6	8	7	Bmc Gastroenterology	2.5
4	9	World Journal of Gastroenterology	4.3	9	6	Digestive Diseases and Sciences	2.5
5	8	Journal of Gastroenterology and Hepatology	3.7	10	6	Frontiers in Neuroscience	3.2

**Figure 3:**
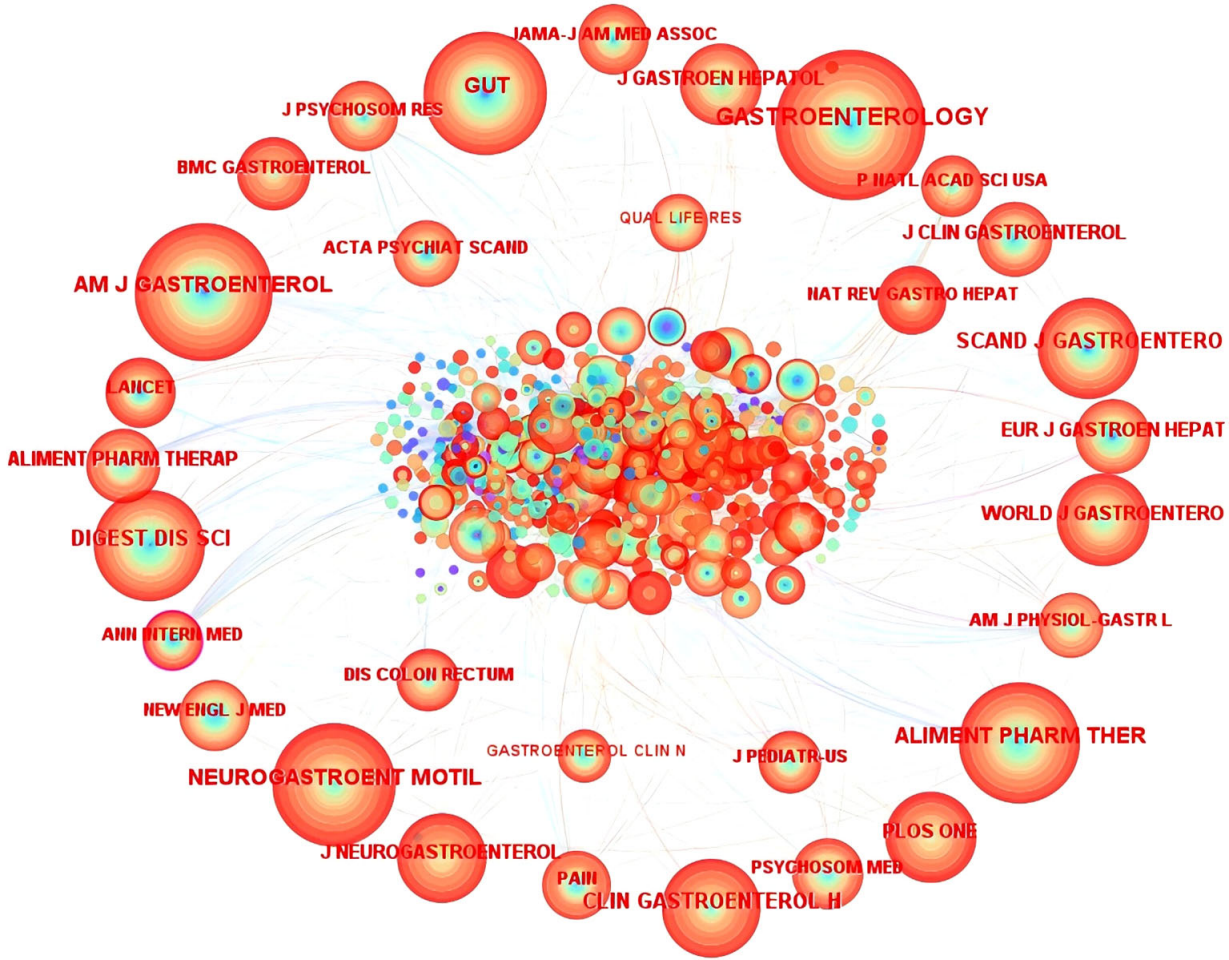
Cited journal map between 2003 and 2024.

CiteSpace software was used to draw a network diagram of cited journals based on co-citation and centrality (**Figure 3**, **Table 4**). The findings showed *Gastroenterology* to have the highest citation frequency, and *Annals of Internal Medicine* to have the highest centrality. This suggests that these two journals have considerable academic influence and authority in this field, and can provide substantial supportive evidence and professional insights for researchers.

**Table 4:** Top 10 cited journals and centrality, as identified by network analysis.

Rank	Cited Journal	Frequency	Rank	Cited Journal	Centrality
1	Gastroenterology	291	1	Annals of Internal Medicine	0.15
2	American Journal of Gastroenterology	246	2	Biological Psychiatry	0.09
3	Gut	197	3	Brain Research	0.09
4	Alimentary Pharmacology Therapeutics	195	4	Lancet	0.08
5	Neurogastroenterology and Motility	195	5	American Journal of Physiology–Gastrointestinal and Liver Physiology	0.08
6	Digestive Diseases and Sciences	169	6	Journal of Pediatrics	0.08
7	Scandinavian Journal of Gastroenterology	131	7	Proceedings of The National Academy of Sciences of The United States of America	0.08
8	Clinical Gastroenterology and Hepatology	124	8	Pain	0.07
9	World Journal of Gastroenterology	114	9	American Journal of Psychiatry	0.06
10	Plos One	108	10	Journal of Psychosomatic Research	0.06

### 3.3 Analysis of countries and institutions

**Figure 4:**
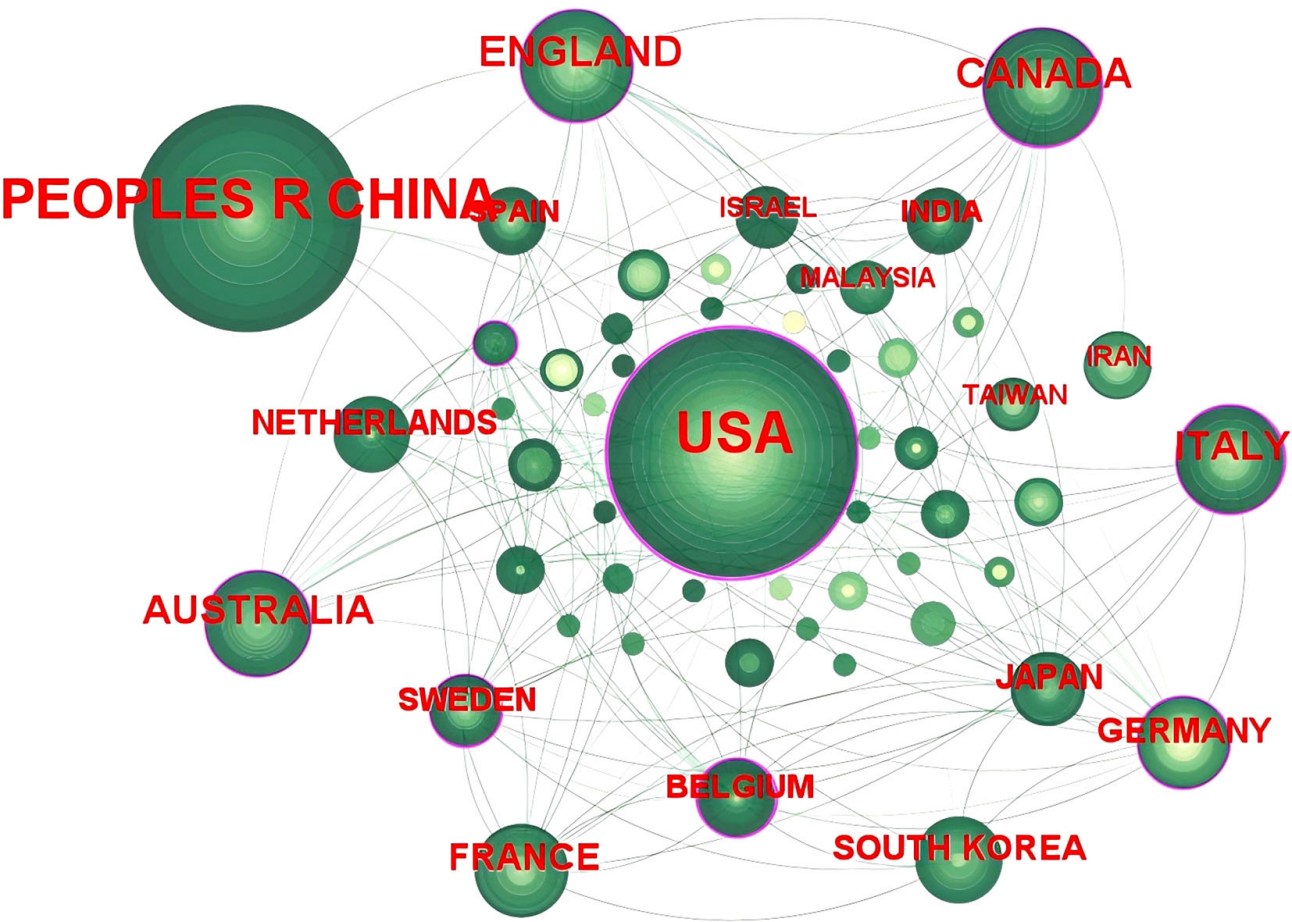
Map of countries that published research on FC with anxiety or depression between 2003 and 2024.

Analysis of publishing countries helps demonstrate the primary distribution of global research. A distribution map of the country partnership network was generated using CiteSpace software; the network comprised 53 nodes and 163 connectors (**Figure 4**), representing 427 articles from 53 countries. Most journals were published in the United States of America (USA) (135, 31.61%); it was followed by the People’s Republic of China (105, 24.59%), Canada (33, 7.73%), England (30, 7.03%), and Australia (28, 6.56%), in that order. The highest centrality was found in the USA (0.58), followed by Belgium (0.24), Germany (0.16), Australia (0.14), and Canada (0.14), in that order; the 10 major countries in terms of journal publication and centrality are shown in **Table 5**. As seen in **Figure 4** and **Table 5**, the USA has conducted extensive and high-quality research on FC with anxiety or depression; this research is the most representative and influential, and provides a substantial theoretical basis for researchers.

**Table 5:** Top 10 publications and centrality of countries, as identified by network analysis.

Rank	Country	Publications	Rank	Country	Centrality
1	USA	135	1	USA	0.58
2	People’s Republic of China	105	2	Belgium	0.24
3	Canada	33	3	Germany	0.16
4	England	30	4	Australia	0.14
5	Australia	28	5	Canada	0.14
6	Italy	25	6	England	0.14
7	France	20	7	Singapore	0.13
8	Germany	17	8	Sweden	0.11
9	South Korea	16	9	Italy	0.10
10	Japan	13	10	Japan	0.09

**Figure 5:**
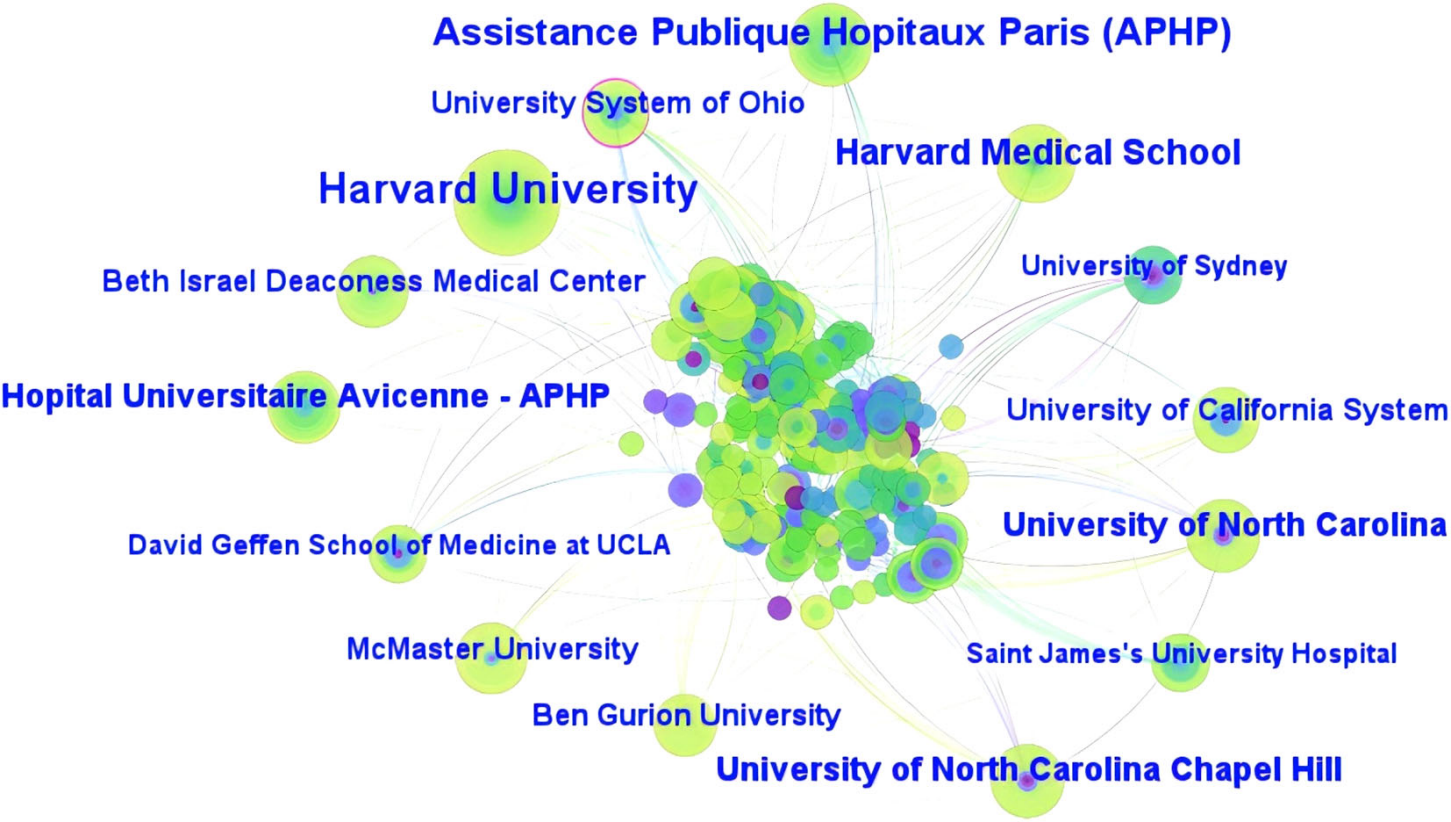
Map of institutions with published research on FC with anxiety or depression between 2003 and 2024.

Research institutions are centers for scientific research. Analysis of the literature published by different institutions can therefore help understand the distribution of the main research groups in the field. As shown in **Figure 5**, 427 articles were published by 365 research institutions. The institution with the highest number of published articles was Harvard University (21, 4.92%); it was followed by the Assistance Publique-Hôpitaux de Paris, University of North Carolina and University of North Carolina Chapel Hill (15, 3.51% each), and University Paris Cite (13, 3.04%), in that order. The University System of Ohio (0.15) demonstrated the highest centrality, followed by McMaster University and the University of Amsterdam (0.10 each), and Saint James’s University Hospital and Columbia University (0.08 each), in that order. **Table 6** shows the top 10 institutions in terms of article publication volume and centrality. The research institutions mainly included comprehensive universities such as the Harvard University and University of North Carolina. The participation of medical centers or those related to a specific discipline was relatively low; this may be attributed to the abundance of academic resources and better faculty in comprehensive universities.

**Table 6:** Top 10 publications and centrality of institutions, as identified by network analysis.

Rank	Publications	Institutions	Rank	Centrality	Institutions
1	21	Harvard University	1	0.15	University System of Ohio
2	15	Assistance Publique Hopitaux Paris(APHP)	2	0.10	McMaster University
3	15	University of North Carolina	3	0.10	University of Amsterdam
4	15	University of North Carolina Chapel Hill	4	0.08	Saint James's University Hospital
5	13	University Paris Cite	5	0.08	Columbia University
6	13	University System of Ohio	6	0.08	Cornell University
7	13	Harvard Medical School	7	0.07	KU Leuven
8	12	Hopital Universitaire Avicenne – APHP	8	0.07	University of Nottingham
9	12	McMaster University	9	0.06	Harvard University
10	11	University of California System	10	0.05	Assistance Publique Hopitaux Paris(APHP)

### 3.4 Analysis of publishing and cited authors

**Figure 6:**
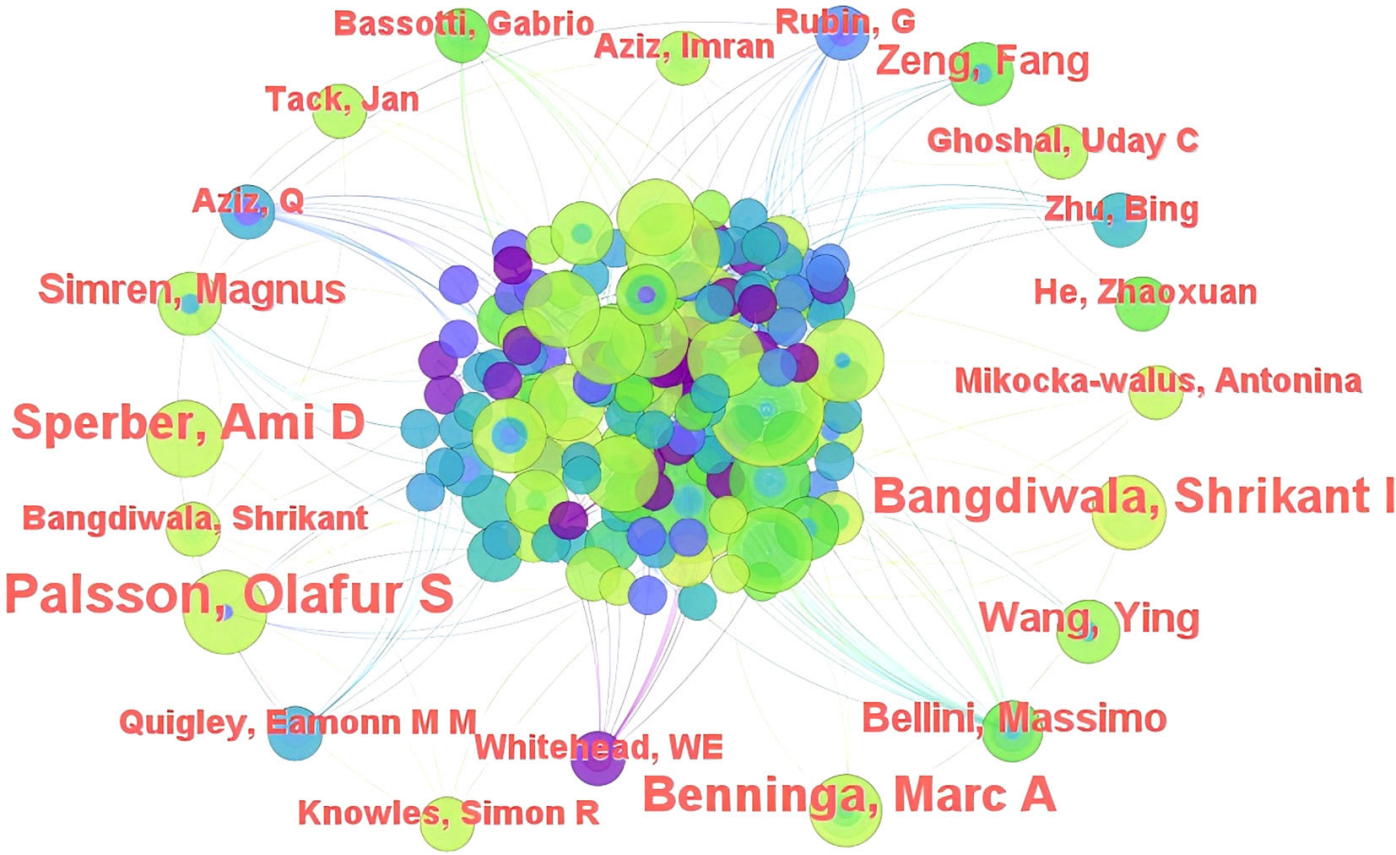
Map of authors who researched the area of FC with anxiety or depression between 2003 and 2024.

Analysis of data pertaining to publishing and cited authors can help identify the major contributors in a certain research field. Data regarding all participating authors of these 427 articles were imported into CiteSpace for analysis, and a collaborative network map was generated (**Figure 6**). This map not only showed the authors who made the greatest contributions in the field, but also helped understand the degree of collaboration between different authors.

The author with the highest number of publications on FC with anxiety or depression was found to be Michel Bouchoucha (13 articles); he was followed by Robert Benamouzig (12 articles), Prashant Singh (9 articles), Shrikant I Bangdiwala (8 articles), and Nicholas J Talley (7 articles), in that order. The top 10 authors are shown in **Table 7**. Comprehensive analysis showed that although Michel Bouchoucha was the highest contributor in this research field, he did not widely collaborate with authors from different teams. This may be attributed to differences in research directions among various research groups.

**Table 7:** Top 10 authors with publications related to FC with anxiety or depression.

Rank	Publications	Author	Rank	Publications	Author
1	13	Bouchoucha, Michel	6	7	Ford, Alexander
2	12	Benamouzig, Robert	7	7	Iturrino, Johanna
3	9	Singh, prashant	8	7	Nee, Judy
4	8	Bangdiwala, Shrikant I	9	7	Palsson, O. S
5	7	Talley, Nicholas J	10	6	Devroede, Ghislain

**Figure 7:**
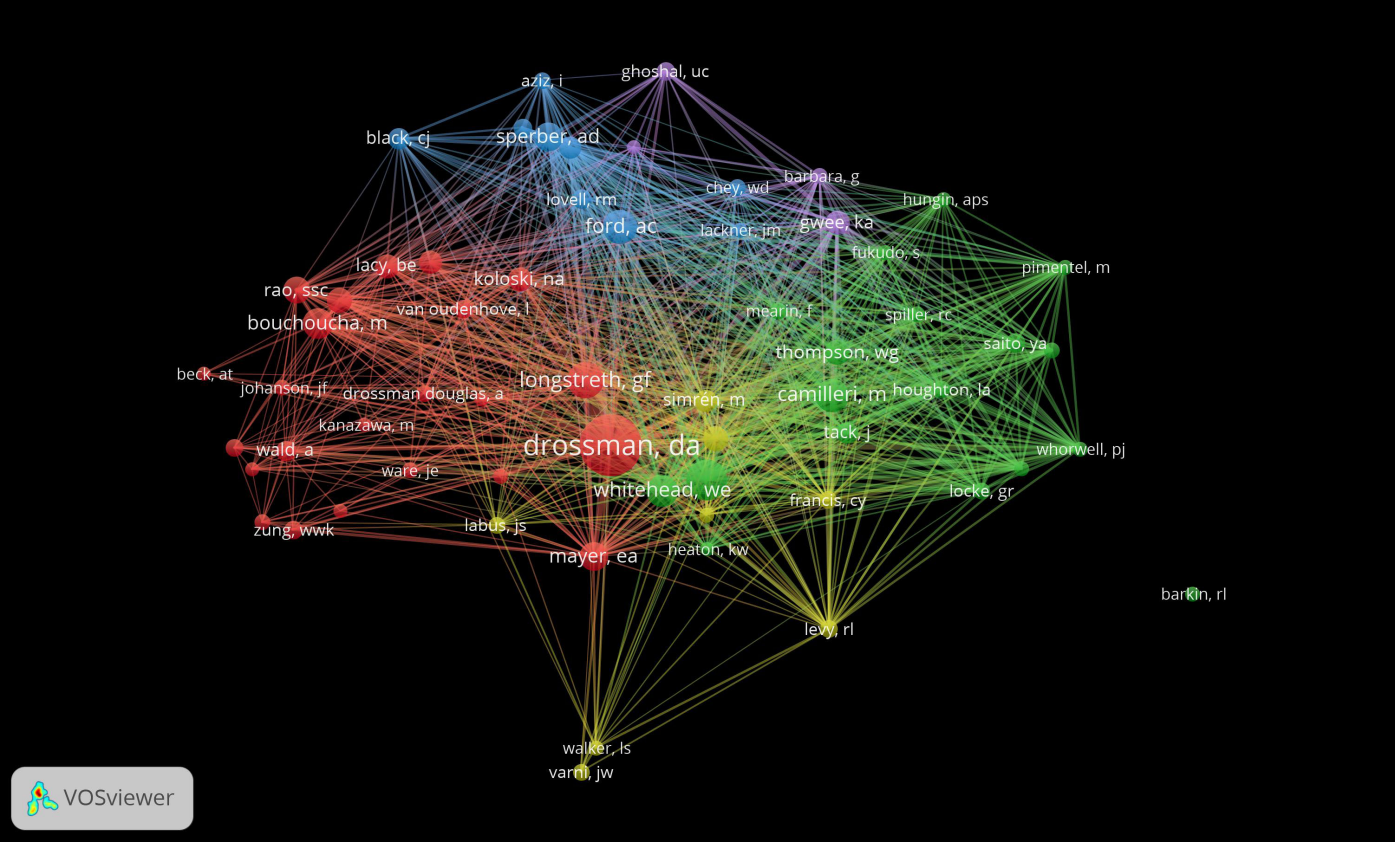
Map of cited authors with published research on FC with anxiety or depression between 2003 and 2024.

Co-citation analysis can help identify the core authors and their connections in the field (**Figure 7**). The most cited author was DA Drossman (153, 35.83%), who was followed by GF Longstreth (88, 20.61%), NJ Talley (73, 17.10%), M Camilleri (57, 13.35%), and WE Whitehead (55, 12.88%), in that order; L Chang demonstrated the highest centrality (0.18), followed by GF Longstreth (0.11), DA Drossman and NA Koloski (0.09 each), and M Camilleri (0.08). **Table 8** shows the top 10 cited authors in terms of frequency and centrality. In summary, DA Drossman and L Chang were identified to be active researchers in the field; they are performing extensive research and are making outstanding academic contributions.

**Table 8:** Top 10 cited authors in terms of frequency and centrality.

Rank	Frequency	Author	Rank	Centrality	Author
1	153	Drossman, DA	1	0.18	Chang, L
2	88	Longstreth, GF	2	0.11	Longstreth, GF
3	73	Talley, NJ	3	0.09	Drossman, DA
4	57	Camilleri, M	4	0.09	Koloski, NA
5	55	Whitehead, WE	5	0.08	Camilleri, M
6	54	Ford, AC	6	0.08	Aziz, I
7	47	Sperber, AD	7	0.07	Whitehead, WE
8	45	Lacy, BE	8	0.07	Bharucha, AE
9	42	Mayer, EA	9	0.06	Levy, RL
10	40	Zigmond, AS	10	0.05	Talley, NJ

### 3.5 Analysis of cited references

**Figure 8:**
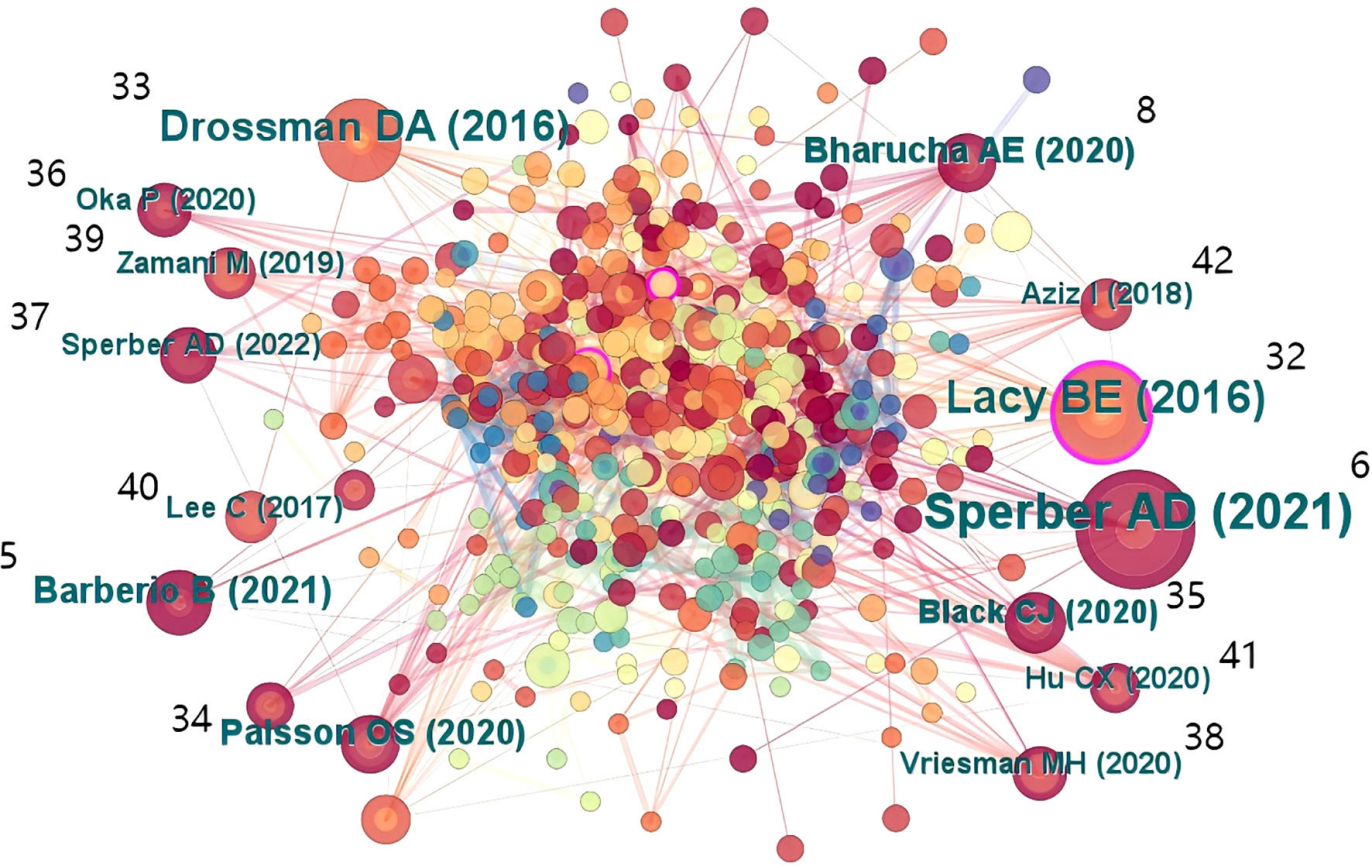
Map of cited references related to FC with anxiety or depression between 2003 and 2024.

The co-occurrence chart of references can help identify high-quality literature related to FC with anxiety or depression. This is useful for researchers, as it may help in rapid identification of research hotspots and frontiers in the field (32–42). The references are labeled in **Figure 8**.

**Table 9** shows the top 10 references with the highest citation frequency; these are highly influential and representative within this research field. Centrality is a pivotal metric for assessing the significance of node position in the network. Nodes with a centrality of more than 0.1 are usually deemed to be critical. The nodes with high centrality are denoted by purple halos in the table; this can aid in the rapid identification of the most valuable nodes in the network. **Table 10** shows the rankings of the top 10 references in terms of centrality; it indicates the references that have a higher impact and influence in the research field.

**Table 9:** Top 10 cited references in terms of frequency.

Rank	Frequency	References	Author and publication year
1	36	Gastroenterology, V160, P99. DOI 10.1053/j.gastro.2020.04.014 (6)	Sperber AD, 2021
2	24	Gastroenterology, V150, P1393. DOI 10.1053/j.gastro.2016.02.031 (32)	Lacy BE, 2016
3	18	Gastroenterology, V150, P1262. DOI 10.1053/j.gastro.2016.02.032 (33)	Drossman DA, 2016
4	12	Lancet Gastroenterol, V6, P638. DOI 10.1016/S2468–1253(21)00111–4 (5)	Barberio B, 2021
5	10	Gastroenterology, V158, P1262. DOI 10.1053/j.gastro.2019.12.021 (34)	Palsson OS, 2020
6	10	Gastroenterology, V158, P1232. DOI 10.1053/j.gastro.2019.12.034 (8)	Bharucha AE, 2020
7	9	Lancet, V396, P1664. DOI 10.1016/S0140–6736(20)32115–2 (35)	Black CJ, 2020
8	8	Lancet Gastroenterol, V5, P908. DOI 10.1016/S2468–1253(20)30217–X (36)	Oka P, 2020
9	8	Clin Gastroenterol H, V20, PE945. DOI 10.1016/j.cgh.2021.05.042 (37)	Sperber AD, 2022
10	8	Nat Rev Gastro Hepat. V17, P21. DOI 10.1038/s41575–019–0222–y (38)	Vriesman MH, 2020

**Table 10:** Top 10 cited references in terms of centrality.

Rank	Centrality	References	Author and publication year
1	0.19	Gastroenterology, V150, P1393. DOI 10.1053/j.gastro.2016.02.031 (32)	Lacy BE, 2016
2	0.13	Aliment Pharm Ther, V37, P555. DOI 10.1111/apt.12208 (43)	Anggiansah R, 2013
3	0.12	Scand J Gastroentero, V48, P523. DOI 10.3109/00365521.2013.775328 (44)	Krogsgaard LR, 2013
4	0.11	Gastroenterology, V150, P1262. DOI 10.1053/j.gastro.2016.02.032 (33)	Drossman DA, 2016
5	0.11	Aliment Pharm Ther, V31, P874. DOI 10.1111/j.1365–2036.2010.04237.x (45)	Barrett JS, 2010
6	0.09	Gastroenterology, V158, P1262. DOI 10.1053/j.gastro.2019.12.021 (34)	Palsson OS, 2020
7	0.09	Aliment Pharm Ther, V33, P1215. DOI 10.1111/j.1365–2036.2011.04640.x (46)	Aro P, 2011
8	0.08	Gut, V61, P997. DOI 10.1136/gutjnl–2011–301501 (47)	Jeffery IB, 2012
9	0.07	J Clin Immunol, V30, P74. DOI 10.1007/s10875–009–9342–4 (48)	Barkhordari E, 2010
10	0.06	Gastroenterology, V160, P99. DOI 10.1053/j.gastro.2020.04.014 (6)	Sperber AD, 2021

**Figure 9:**
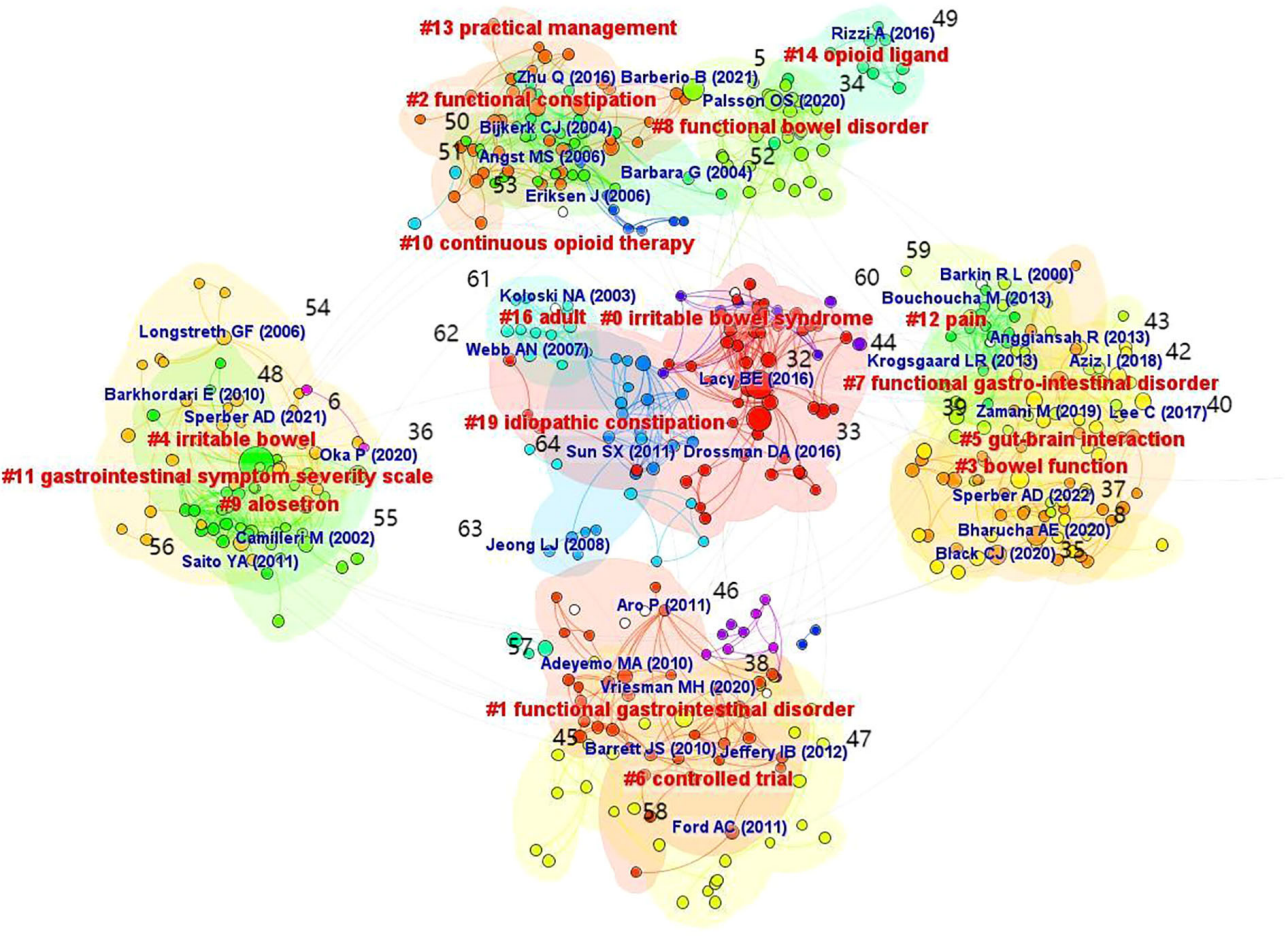
Cluster analysis map of co–citation references related to FC with anxiety or depression between 2003 and 2024.

CiteSpace software uses the log-likelihood ratio algorithm to generate clustering labels for the cluster analysis map, which demonstrates the relationship between cited literature (43–64). This helps identify the research focus and knowledge structure in the field. The references are labeled in **Figure 9**; the results indicated a good cluster structure, as evidenced by the cluster modulus value of 0.9245 (values of > 0.3 are considered to indicate good structure). The average contour value was found to be 0.9591; in this context, values of > 0.7 show clustering to be feasible and reasonable. Based on the above findings, the research related to FC with anxiety or depression was deemed to have high credibility.

As seen in **Figure 9**, a total of 16 clusters were formed; among these, “gut-brain interaction” and “continuous opioid therapy” were the 2 important clusters. This shows that dysfunctions in gut brain interaction and continuous opioid therapy are closely related to FC with anxiety or depression. Notably, studies have shown disorders in gut-brain interaction to be an important factor in the pathogenesis of FC; these usually lead to social-psychological disorders such as anxiety and depression, and a decline in the quality of life. The severity is directly proportional to the number of involved regions (of gut-brain interaction disorders) (37). In this context, patients receiving continuous opioid therapy often experience gastrointestinal symptoms such as constipation and abdominal distension, and have psychological disorders such as anxiety and depression (65).

### 3.6 Analysis of keywords

**Figure 10:**
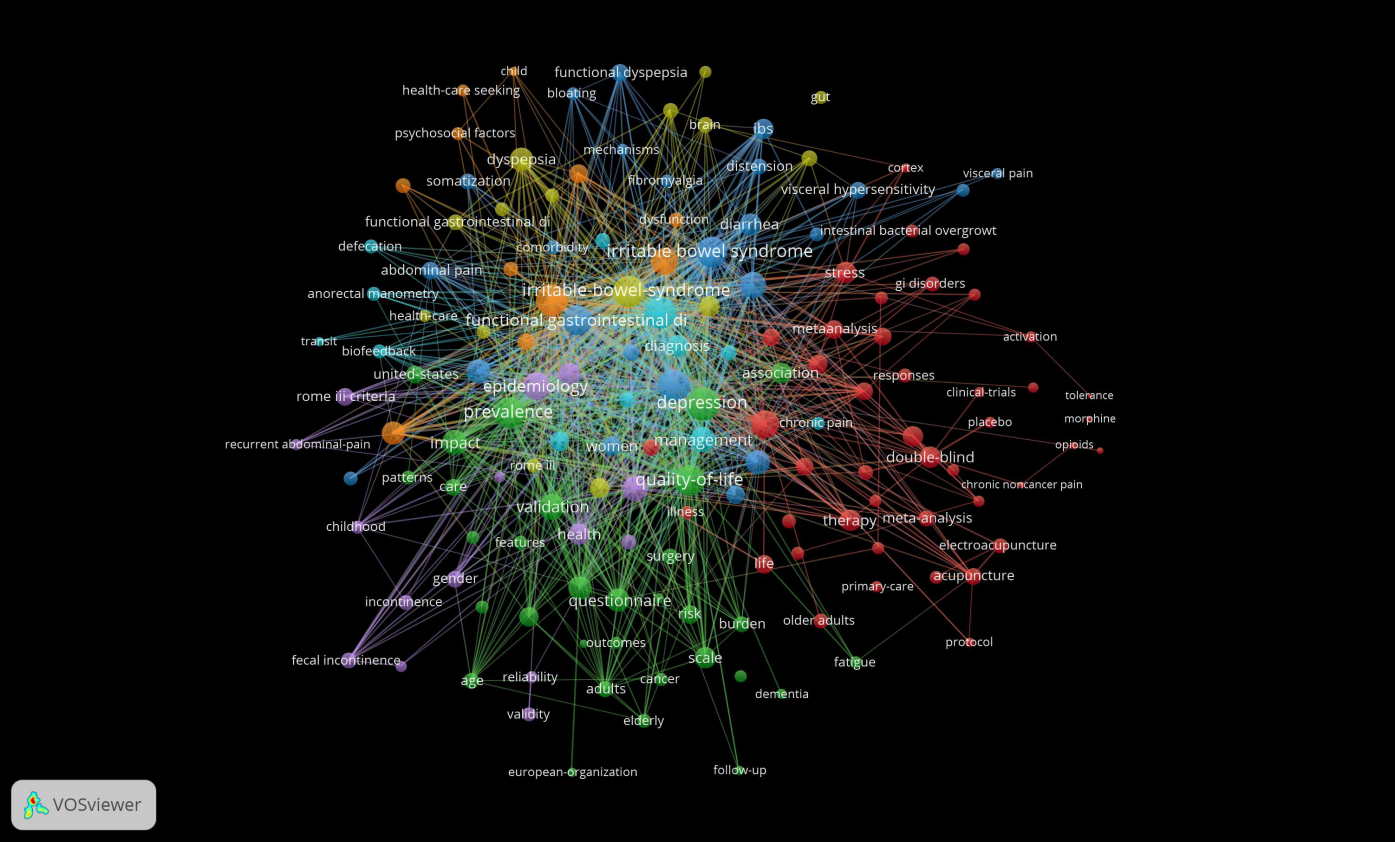
Map of keywords related to FC with anxiety or depression between 2003 and 2024.

**Figure 11:**
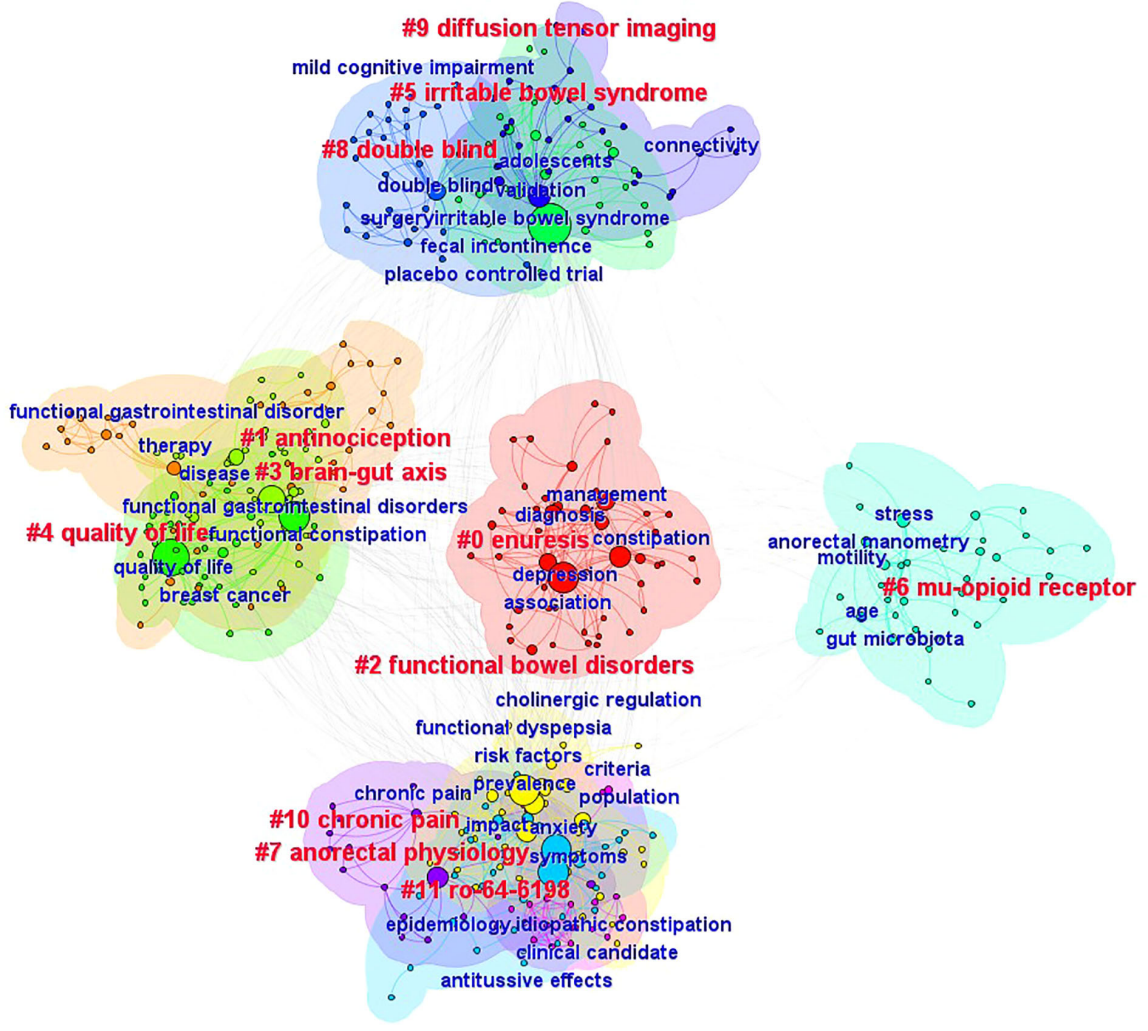
Cluster analysis map of co–citation keywords related to FC with anxiety or depression between 2003 and 2024.

Keywords represent the central idea of the paper and summarize the theme of the article. Co-occurrence analysis of keywords therefore helps identify the core viewpoints and popular topics in a particular research field (**Figure 10**). In this study, “irritable bowel syndrome,” “quality of life,” “prevalence,” “symptoms,” and “depression” were found to be the most popular keywords (**Table 11**). Notably, keyword clustering can summarize the principal research clusters in a certain research field to some extent. A total of 12 clusters were identified in this study using CiteSpace software (**Figure 11**). The cluster modulus and average contour values of 0.5206 and 0.7943, respectively, showed the clustering to be reasonable and feasible.

**Table 11:** Top 10 keywords in terms of frequency and centrality.

Rank	Frequency	Keyword	Rank	Centrality	Keyword
1	155	irritable bowel syndrome	1	0.27	quality of life
2	114	quality of life	2	0.17	irritable bowel syndrome
3	83	prevalence	3	0.16	double blind
4	81	symptoms	4	0.15	functional gastrointestinal disorders
5	79	depression	5	0.13	constipation
6	77	functional gastrointestinal disorders	6	0.13	gastrointestinal disorders
7	69	anxiety	7	0.13	children
8	56	functional constipation	8	0.13	activation
9	43	disorders	9	0.12	symptoms
10	39	constipation	10	0.11	depression

Among the clusters, “mu-opioid receptor” and “chronic pain” were identified to be the most important. This indicates that the research hotspots for FC with anxiety or depression may be related to the etiology and pathogenesis of the condition. Patients with chronic pain who rely on opioid therapy often experience opioid-induced FC; in addition, the prevalence of anxiety and depression increases with an increase in opioid dosage (66). In this context, opioids (such as morphine, oxycodone, and fentanyl) are powerful analgesic drugs that are widely used in the clinic. These drugs exert analgesic, anesthetic, hypnotic, and other pharmacological effects which are mainly mediated via interactions with opioid receptors in the brain (67). Despite their widespread use, they are associated with various side effects; in addition to euphoria and analgesia, these drugs cause other serious side effects such as constipation and respiratory depression (68). Notably, opioid abuse is currently a major public health problem worldwide (69). The prevalence of opioid intake for functional gastrointestinal diseases has increased over the past few years; this is associated with a higher incidence of gastrointestinal side effects (such as constipation, abdominal distension, and vomiting) and depression, and changes in the quality of life (65).

**Figure 12:**
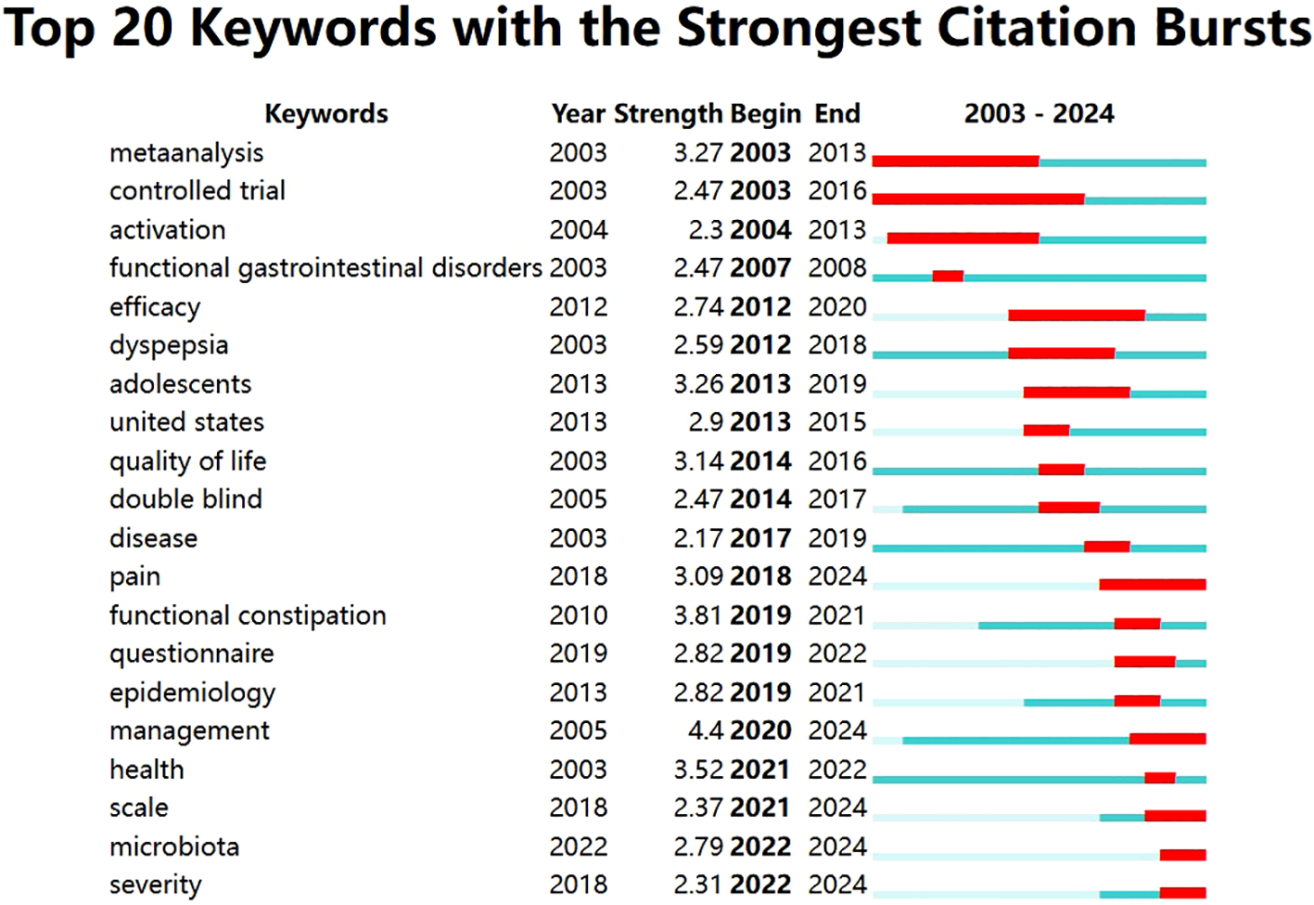
Top 20 keywords with the strongest citation bursts related to FC with anxiety or depression.

Burst detection usually includes the detection of both intensity and chronological distribution of keywords that appear frequently and demonstrate a high growth rate over a period of time. It can indicate the research dynamics of a research field over time and predict future research trends. The top 20 keywords with the strongest citation bursts between 2003 and 2024 are shown in **Figure 12**. The identified burst words indicate considerable diversity in research perspectives during this time; they mainly demonstrate the research projects and diseases related to FC with anxiety or depression. Most studies were conducted in the USA, and the research projects mainly included randomized double-blind controlled trials and methodological design studies (such as meta-analyses and evaluation of questionnaires and scales). **Figure 12** also shows that FC with anxiety or depression is closely related to dyspepsia. Patients with FC tend to develop overlapping symptoms of dyspepsia; in particular, those with a history of anxiety and depression are more likely to demonstrate overlapping symptoms (70).

**Figure 13:**
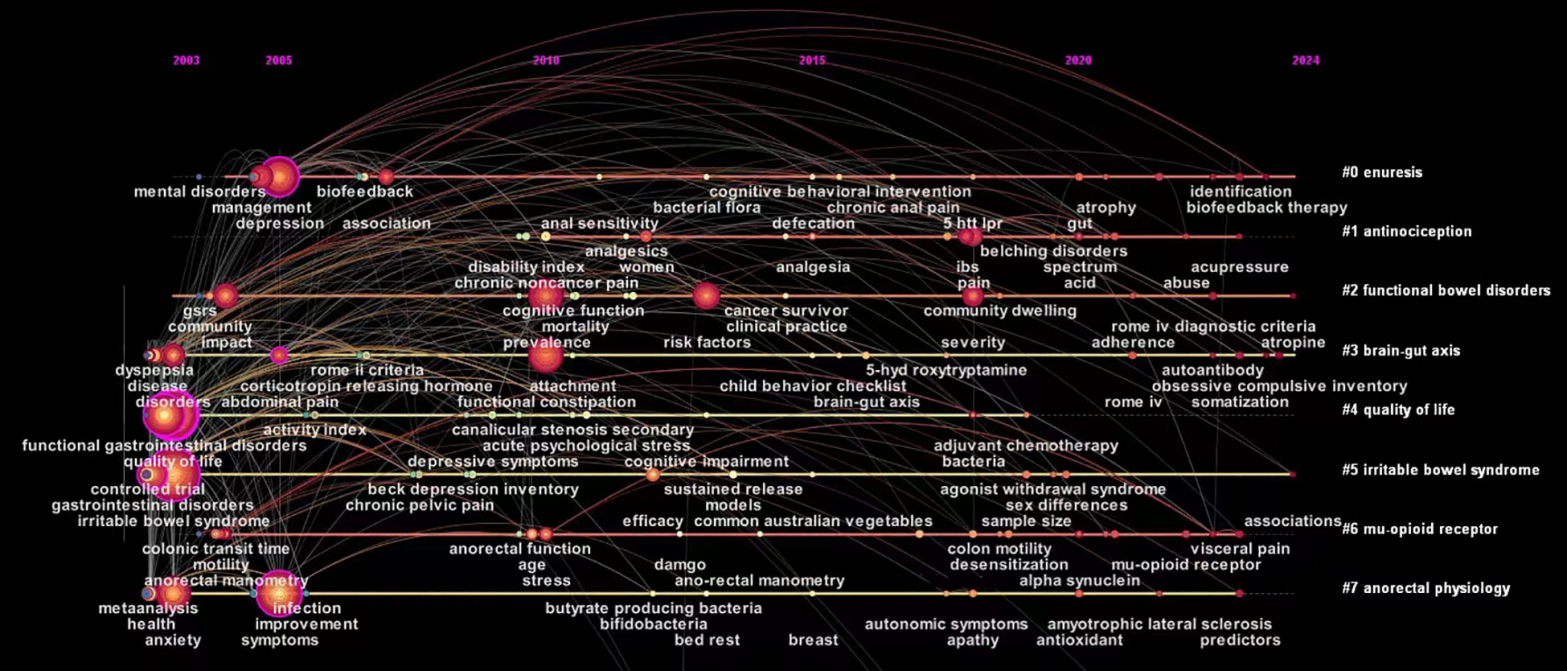
Timeline map of keywords related to FC with anxiety or depression.

The temporal changes in keywords across different clusters were also evaluated in this study. The clusters (to which the nodes belonged) were plotted on the vertical axis and the publishing time (2003 – 2024) was plotted on the horizontal axis, each node was reasonably distributed in the corresponding position, and a visual keyword clustering timeline was generated (**Figure 13**). This could directly show the evolution of research hotspots. The findings showed that in recent years, the treatments for FC with anxiety or depression involve the use of bacterial flora and biofeedback therapy. In this context, the intestinal flora refers to the microbial community in the human gastrointestinal tract, that is considered to be the potential common pathophysiological basis of functional gastrointestinal disorders and mental diseases (such as depression and anxiety) (71). Studies have shown that the numbers of *Bacteroides*, *Rosella*, and *Faecococcus* decrease in patients with FC (72, 73), and those of actinobacteria correlate negatively with the severity of depression and anxiety (15). Studies have also shown that fecal microbiota transplantation can relieve symptoms of constipation, depression, and anxiety by rebalancing the intestinal flora (18, 70, 74). Biofeedback therapy involves the measurement of anal pressure or electromyography, that enables patients to intuitively perceive changes in pressure in the pelvic floor muscles and rectum. Patients are then trained to relax the pelvic floor and external anal sphincter, increase intra–abdominal pressure, and adjust the coordination between abdominal and anorectal muscles to relieve constipation (75, 76). Biofeedback therapy is a safe and effective treatment for FC (77). Studies have shown that it can improve clinical symptoms, psychological status, and quality of life in affected patients (78).

As seen in **Figure 13**, clustering of the brain–gut axis demonstrated the widest time span, with nodes distributed between 2003 and 2024. Notably, the node distribution has been relatively dense in recent years, this indicates that the brain–gut axis is currently an area of particular interest in research on FC with anxiety or depression. This axis plays a key role in the pathogenesis of functional gastrointestinal diseases. According to the Rome IV diagnostic criteria, gastrointestinal diseases caused by abnormal brain–gut interactions are classified as functional (79). Notably, abnormal interactions also represent a key factor in the development of psychological disorders in patients with long–term constipation. Studies show that up to 65% of patients with chronic constipation have different degrees of mental illnesses such as anxiety or depression (80). In addition, the brain–gut axis has been found to play a two–way role in the relationship between mental disorders and gut microecology. Findings also suggest that the intestinal flora have a regulatory effect on anxiety, emotion, and cognition, and these effects are exerted via the gut–brain axis (81, 82).

## 4 Conclusion

This study included 427 articles (retrieved from the Web of Science Core Collection database) on FC with anxiety or depression that were published between 2003 and 2024. Bibliometric methods were used in addition to CiteSpace, VOSviewer, and Excel software to organize and summarize the data and draw visual knowledge maps. This helped explore the current research status, hotspots, and developmental trends in the field of FC with anxiety or depression, it also helped analyze existing problems, with the aim of providing new ideas for future research.

The key findings indicated a slight fluctuation in the quantity of articles related to FC with anxiety or depression between 2003 and 2024. However, an overall upward trend was identified, this suggested that an increasing number of researchers are focusing on this research domain. The 427 articles included 6 types of publications, of which original research articles accounted for the highest proportion (357 articles, 83.61%). These were published across 200 journals, of which *Neurogastroenterology and Mobility* had the highest number of articles (30, 7.02%), and *The Lancet* had the highest impact factor (98.4 in 2023). The USA was found to be the country with the most published papers (135 articles, 31.61%), and Harvard University was identified as the research institution with the most published papers (21 articles, 4.92%), Michel Bouchoucha was found to be the most published author (13 articles, 3.04%).

Keywords are highly summarized descriptions of the theme and content of an article. The analysis of keywords may therefore provide suggestions for future research hotspots and frontiers. Our findings show that in the area of basic medicine, the etiology and pathogenesis of FC with anxiety or depression warrants further investigation, with particular focus on opioid drugs (as key etiological agents). Studies also need to evaluate the role of the brain–gut axis in the pathogenesis of this condition. In clinical medicine, emphasis needs to be placed on the design of evidence–based methodology, especially randomized double–blind controlled trials with stringent criteria, high–quality meta–analyses, and evaluation of questionnaires and scales. Treatment techniques need to be explored in further detail. In this context, fecal microbiota transplantation and biofeedback therapy need to be investigated further. As FC with anxiety or depression is closely related to dyspepsia, patients with FC tend to have overlapping symptoms of dyspepsia, in particular, those with a history of anxiety and depression are more likely to have overlapping symptoms.

This study also identified several issues in the research field. Most studies have been performed in the USA and China, this may be related to the national economy and level of healthcare. Other countries therefore need to focus on the development of medical technology. The research institutions were found to mainly include comprehensive universities. This may be attributed to the fact that these universities have abundant academic resources and better faculty. Other medical centers or those related to a specific discipline need to place emphasis on academic research. Our findings also showed cooperation and exchange between different scientific research institutions to be limited, this was particularly relevant to international exchange. Increasing exchange between different research institutions and researchers, and improving cooperation across regions, countries, institutions, teams, and specialties, may provide more high–quality and high–impact research. It may also lead to the development of more practical and useful clinical interventions.

The limitations of this study are twofold. First, only the Web of Science database was accessed, which may have introduced certain limitations to the research findings. Future investigations could incorporate complementary databases (e.g., Scopus, EMBASE) to enhance systematicity and comprehensiveness. Second, while CiteSpace and VOSviewer offer extensive data analysis functions, these were not fully utilized in this study. Further exploration of their functionalities to analyze hotspots and frontiers in functional constipation with anxiety or depression is warranted in future research.

## Data Availability

The original contributions presented in the study are included in the article/supplementary material. Further inquiries can be directed to the corresponding author/s.
